# Puerarin: a hepatoprotective drug from bench to bedside

**DOI:** 10.1186/s13020-024-01011-y

**Published:** 2024-10-08

**Authors:** Yi-Xiang He, Meng-Nan Liu, Hao Wu, Qi Lan, Hao Liu, Maryam Mazhar, Jin-Yi Xue, Xin Zhou, Hui Chen, Zhi Li

**Affiliations:** 1 The Key Laboratory of Integrated Traditional Chinese and Western Medicine for Prevention and Treatment of Digestive System Diseases of Luzhou City, Affiliated Traditional Medicine Hospital of Southwest Medical University, Luzhou, 646000 China; 2 Department of Spleen and Stomach Diseases, The Affiliated Traditional Chinese Medicine Hospital of Southwest Medical University, Luzhou, 646000 Sichuan China; 3 Affiliated Traditional Chinese Medicine Hospital, Southwest Medical University, Luzhou, 646000 Sichuan China; 4 Department of Pediatrics, The Affiliated Hospital, Southwest Medical University, Luzhou, 646000 Sichuan China

**Keywords:** Puerarin, Liver disease, Pharmacological effects, Chinese herbal medicine

## Abstract

Pueraria is a time-honored food and medicinal plant, which is widely used in China. Puerarin, the main component extracted from pueraria, has a variety of pharmacological characteristics. In recent years, puerarin has received increasing attention for its significant hepatoprotective effects, such as metabolic dysfunction-associated steatotic liver disease, alcohol-related liver disease, and hepatic carcinoma. This paper explores the pharmacological effects of puerarin on various liver diseases through multiple mechanisms, including inflammation factors, oxidative stress, lipid metabolism, apoptosis, and autophagy. Due to its restricted solubility, pharmacokinetic studies revealed that puerarin has a low bioavailability. However, combining puerarin with novel drug delivery systems can improve its bioavailability. Meanwhile, puerarin has very low toxicity and high safety, providing a solid foundation for its further. In addition, this paper discusses puerarin's clinical trials, highlighting its unique advantages. Given its excellent pharmacological effects, puerarin is expected to be a potential drug for the treatment of various liver diseases.

## Introduction

The liver is the body's main digestive gland and organ for detoxification, with functions such as regulating bile, glucose, and lipids [1]. Common liver diseases include alcohol-related liver disease (ALD), metabolic dysfunction-associated steatotic liver disease (MASLD), drug-induced liver injury (DILI), and hepatocellular carcinoma [2]. The primary causes of liver disease include drug toxicity, alcoholism, viral infections, and malnutrition [1]. According to epidemiology, liver disease accounts for 4% of the disease mortality rate, resulting in approximately 2 million fatalities annually [3]. Over the past 20 years, the number of liver disease-related deaths in the United Kingdom has increased by 63.6%, with male patients accounting for 60% of these deaths [4]. While new vaccines and medications may reduce the prevalence of certain liver illnesses in the developed world, such steps are still restricted in developing nations [5]. Therefore, there is an urgent need to find new drugs to prevent and treat liver disease.

In the last few years, natural active compounds in nature have received increasing attention due to their less toxic and beneficial effects on health. *Pueraria montana* var. lobate (Willd.) Maesen & S.M.Almeida ex Sanjappa & Predeep, the dried root of the perennial *Pueraria lobata* Willd Ohwi or *Pueraria thomsonii* Benth of the genus Pueraria of the family Leguminosae, serves as a food as well as a medicinal plant that was first recorded in the Sheng Nong's Herbal Classic (A.D. 220–280) [6, 7]. Pueraria is mainly found in Sichuan, Zhejiang, and Guangdong, and has the advantages of high production and low price [8]. In traditional use, pueraria significantly relieves muscles, reduces fever, rejuvenates, relieves diarrhea [9], and can treat drunkenness and alcoholism [10]. Modern pharmacology suggests that pueraria has the effects of reducing lipid deposition, dilating blood vessels, inhibiting inflammation, and alleviating hangovers [11], which is broadly in line with traditional uses. Puerarin, the main active ingredient isolated from pueraria, is widely used in treating cardiovascular diseases, diabetes mellitus, and liver diseases [12]. In-depth research on puerarin has found its antioxidant properties and pharmacological effects on immune function and inhibition of inflammation [13, 14]. However, comprehensive reviews of its effects on liver diseases are rare. Therefore, this paper explores the mechanisms of action of puerarin in various liver disorders to provide a theoretical foundation for future study and clinical application.

## Physicochemical properties and bioavailability of puerarin

**Fig. 1:**
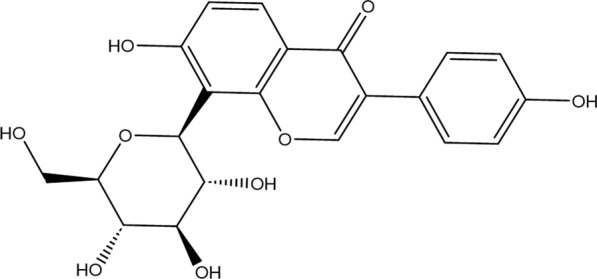
Chemical structure of puerarin

Puerarin is the phytoestrogen and the main biologically active isoflavone [15]. According to the Chinese Pharmacopoeia, the content of puerarin in pueraria should not be less than 13%, expressed as 8-(β-d-Glucopyranosyl)-4′, 7-dihydroxy isoflavone [16]. In puerarin, the bond length of 7–O–H is slightly longer than that of 4′–O–H because of the weak intramolecular hydrogen bond between 7–H and the sugar group. The glycosyl group is in chair conformation, where four hydroxyl groups form three intramolecular hydrogen bonds. The introduction of the glucose moiety makes puerarin strongly hydrophilic, which reduces its solubility in lipids [17]. Puerarin has a molecular weight of 416 and a density of 1.642 g/cm^3^ [18]. The chemical structure of puerarin is shown in Fig. 1.

## Pharmacological effects of puerarin in models of liver disease

**Fig. 2:**
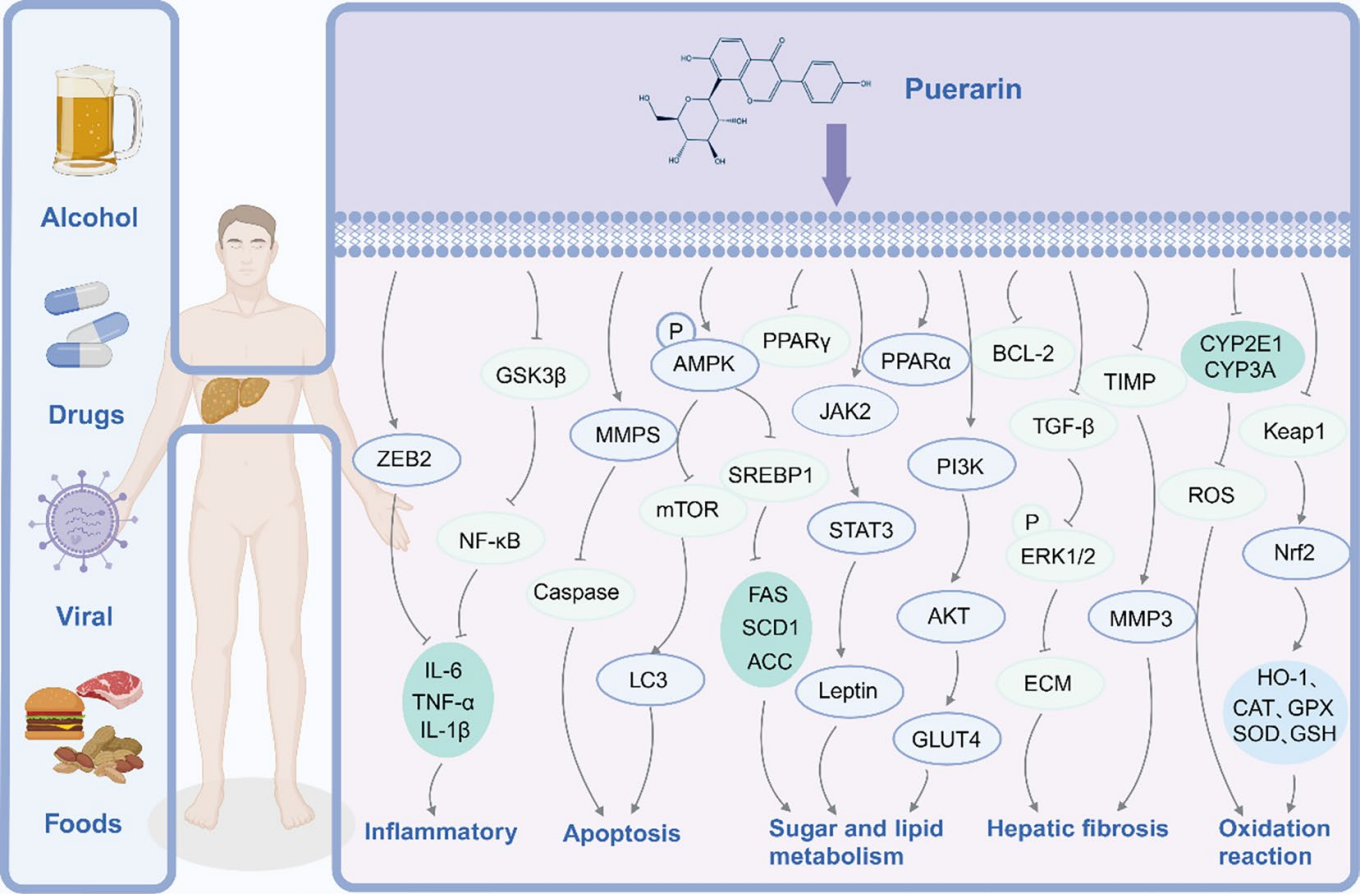
The pharmacological effects of puerarin. Pharmacological effects of Puerarin in liver disease models are mainly related to inflammation, apoptosis, oxidative stress, hepatic fibrosis, sugar and lipid metabolism

The pharmacological effects of puerarin in liver disease models mainly involve inflammation, oxidative stress, steatosis, and apoptosis, as shown in Fig. 2. More details about the pharmacological effects of puerarin in liver disease are shown in Table 1.

**Table 1:** The pharmacological effects of puerarin

Number	Models	Type	Dosage (Puerarin)	Main results	Molecular mechanisms	References
1	NIAAA model	In vivo	100 mg/kg for 10 days	↓ SREBP-1c, PPAR-α, TNF-α, IL-6 and IL-1β	Puerarin inhibits inflammation and lipid accumulation in alcoholic liver disease	[68]
	Ethanol-induced AML-12 cell	In vitro	0, 6.25, 12.5, 25, 50 and 100 μmol-L-1			
2	The rat hepatoma cell line H4IIE	In vitro	30, 60, 120,180, or 240 μM for 24 h	↑ LC3-II, AMPK, mTOR ↓ Lipid concentration	Puerarin restored the viability of cells and reduced lipid accumulation in ethanol-treated hepatocytes	[53]
3	Ethanol-induced acute alcoholic liver injury in Wistar rats	In vivo	200 mg/kg/day for 5 days	↓MDA, SOD, GPX	Puerarin may have the function of inhibiting the oxidative stress induced by acute alcoholism	[63]
4	Acute alcoholic rat model	In vivo	1 mL/100 g or 500 mg per kg	↑ GABAAR α 1, ADH ↓ GABAAR α 4	Puerarin have preventive effects on alcoholism related disorders	[140]
	Chronic alcoholic mouse model		250 mg and 500 mg per kg body for 12 days			
5	Lieber-DeCarli chronic-alcohol feeding model	In vivo	180 mg/kg or 90 mg/kg for 8 weeks	↑ ZO-1 ↓ cd68, KC activation, CD 14, TLR 4, TLR 2, TNF-α	Puerarin inhibition of endotoxin gut leakage, Kupffer cell activation, and endotoxin receptor expression is involved in the alleviation of chronic alcoholic liver injury in rats	[54]
6	Chronic alcohol feeding model	In vivo	0.75 g/kg/day or 1.5 g/kg/day for 8 weeks	↓ TGF-β1, α-SMA	Puerarin reduced the effects of alcoholic liver injury	[141]
7	A 2% ethanol solution was used to establish a model of alcoholic fatty liver disease	In vivo	50 μM, 25 μM or 12.5 μM for 48 h	↓ CYP 2 y3, CYP 3a 65, ADH 8a, ADH 8b, HMGCRB, FXR, TNF-α	Puerarin alleviated alcohol-induced hepatic steatosis in zebrafish larvae by regulating alcohol and lipid metabolism	[8]
8	chronic alcoholic liver damage model	In vivo	1.0 g/kg/day for 2 weeks	↑ PPAR-γ ↓ AST, ALT, 5-Lox, Cox-2, TNF-α	Puerarin exerts the hepatoprotection from chronic alcohol-Induced liver injury	[71]
9	The chronic liver injury model	In vivo	30, 60 and 120 mg/kg/day for 24 weeks	↑ ALB, ADH, ALDH ↓ ALT, AST, ALP, GSK-3β, TNF-α, NF-κB	Puerarin exerts the hepatoprotection against chronic alcohol-induced liver injury in rats	[70]
10	The chronic liver injury model	In vivo	0.3 mmol/kg for 5 weeks	↓ MDA, TNF-α, IL-6, ALT, LDL-C	puerarin effectively alleviate hepatic damage induced by chronic alcohol administration through potential antioxidant, anti-inflammatory, or anti-apoptotic mechanisms	[62]
11	Gao-binge model	In vivo	30 mM, 42 mg/kg for 2 weeks; intravenously [i.v.]	↓ Atg3, Atg7, LC3, P62	The protective effect of puerarin-loaded mesoporous silicon nanoparticles on alcoholic hepatitis through autophagy pathway	[77]
12	The chronic liver injury model	In vivo	30, 60 and 120 mg/kg. d for 24 weeks	↑ ADH, ALDH ↓ CYP2E1, CYP1A2, CYP3A	Puerarin improves metabolic function leading to hepatoprotective effects in chronic alcohol-induced liver injury in rats	[60]
13	HFFD-fed male SD rats	In vivo	0.1% or 0.2% for 20 weeks	↑ Mttp, Cpt1a, Pnpla2, SOD, GSH-Px, CAT ↓ Srebf1, Chrebp, Acaca, Scd1, Fasn, Acacb, Cd36, Fatp5, Degs1, Plin2, Apob100, IL-1β, IL-18, TNF-α	Puerarin ameliorates nonalcoholic fatty liver in rats by regulating hepatic lipid accumulation, oxidative stress, and inflammation	[47]
14	HFHS-fed male C57BL/6 J mice	In vivo	0.2 g/kg/day or 0.4 g/kg/day for 18 weeks	↑ NAD + , Acox 1, Cpt 1, Cox 5a, MCAD ↓ PARP-1, SREBP-1, ACC 1, ACC 2, FAS, 4-HNE	Puerarin protects against high-fat high-sucrose diet-induced non-alcoholic fatty liver disease	[52]
15	HepG2 cells	In vitro	25, 50, 100 µM	↑ PPARα, AMPK ↓ FAS and SREBP, TG, TC	Puerarin ameliorates hepatic steatosis	[34]
16	HFD-fed Male Wistar rats	In vivo	0.4 g/kg or 0.8 g/kg for 4 weeks	↑leptin receptor, P-JAK2/P-STAT3 ↓TG, TC	Therapeutic effect of puerarin on non-alcoholic rat fatty liver by improving leptin signal transduction	[43]
17	HFFD-fed male Sprague–Dawley rats	In vivo	0.2% for 16 weeks	↑ Phosphorylation of AMPK and ACC, GLUT4, PI3K/Akt ↓ SREBP-1c, FAS, SCD-1 and HMGCR	Puerarin improves hepatic glucose and lipid homeostasis	[42]
	HepG2 cells	In vitro	75 μM and 150 μM for 24 h			
18	HFD-fed female ICR mice	In vivo	0.2%, 0.4% or 0.8%	↑ p-AMPK, HSL, p-HSL, CAT, AMPK, ACO ↓ TC, TG, PPARγ 2, FAS	Puerarin affects lipid accumulation and metabolism in high-fat diet-fed mice	[35]
19	HFD-fed male C57BL/6J	In vivo	200 mg/kg BW for 4 weeks	↑ GSH-Px, CAT, SOD, SLC 7A 11, GPX 4, SIRT 1, p-Nrf 2, Nrf 2, HO-1 ↓ ALT, AST, TG, TC, MDA, Fe2 + , TNF-α, IL-1β, IL-6	Puerarin ameliorates metabolic dysfunction-associated fatty liver disease by inhibiting ferroptosis and inflammation	[142]
	Palmitic acid-induced AML12 cells	In vitro	75 μM			
20	HepG2 hepatocytes	In vitro	5–50 μM	↑ ATGL, AMPK, GPER, SIRT 1 ↓ FASN, SREBP-1c, FREBP	Puerarin attenuates hepatic steatosis	[37]
21	TAA-induced liver fibrosis in male SD rats	In vivo	0.1 ml/10 g for 4 weeks	↓ TGFβ1, α-SMA, collagen type I, fibronectin, p-ERK1/2	Puerarin alleviates liver fibrosis	[94]
22	CCl4-induced liver fibrosis in male C57BL/6J mice	In vivo	100 and 200 mg/kg intraperitoneally once daily for four weeks	↓ α-SMA, collagen-1, TGF-β, CTGF, NF-κB, ROS, PARP-1	Puerarin protects against CCl4-induced liver fibrosis in mice	[90]
23	CCl4-induced liver fibrosis in male Wistar rats	In vivo	200,400, and 800 mg/kg for 60 days	↑ SOD ↓ ALT, AST, Alb, TP, TNF-α, NF-κB, MDA, TGF-βl, iNOS	Puerarin protects against CCl4-induced hepatic fibrosis rats	[85]
24	DMN-induced liver fibrosis in male SD rats	In vivo	800 mg/kg for 4 weeks	↑ smad7, MMP-1 ↓ ALT, AST, HA, LN, PCIII, CIV, Hyp, Col I, TGF-β l, smad2, smad3, α-SMA, TIMP-1	Puerarin, protects against hepatotoxicity thereby leading to anti-fibrotic effect	[100]
25	CCl4-induced liver fibrosis in male Wistar rats	In vivo	0.2, 0.4, 0.8 g/kg for 60 days	↑albumin, T-protein, PPAR-γ, MMP-2 ↓ALT, AST, T-bilirubin, ECM, Hyp, PCIII, Col I, TIMP-1, PI3K, p-Akt	Puerarin has an antifibrotic effect	[86]
26	CCl4-induced liver fibrosis in Male Wistar rats	In vivo	0.4 and 0.8 g/kg i.g., daily for 4 weeks	↓ ALT, AST, bcl-2, HSC	Reversal of chemical-induced liver fibrosis in Wistar rats by puerarin	[101]
27	CCl4-induced liver fibrosis in male Wistar rats	In vivo	0.4 g/kg for 8 weeks	↓ HA, Hyp, Collagen I, Collagen III, Wnt, β-catenin	Puerarin is capable of alleviating CCl4-induced hepatic fibrosis of rats	[84]
28	SMMC7721 human hepatocellular carcinoma cell	In vitro	400, 800, 1600, 3200, 6400 μM for 48 h	↑Apoptosis ↓ The proliferation of SMMC7721 cells	The effect of puerarinon hepatocellular carcinoma	[104]
29	SMMC-7721 human hepatocellular carcinoma cells	In vitro	500, 1000 and 1500 μg/mL for 12 or 24 h	↑ Caspase-3, caspase-8, caspase-9, AIF, P38, ERK, c-Jun N	Puerarin inhibits growth and induces apoptosis in SMMC-7721 hepatocellular carcinoma cells	[106]
30	SMMC-7721 human hepatocellular carcinoma cells	In vitro	0, 50, 100, 250, 500, 1000, 1500, 2000 μg/m for 12, 24 or 48 h	↑ Apoptosis, MAPK, P-MAPK ↓ The proliferation of SMMC7721 cells	Puerarin induces hepatocellular carcinoma cell apoptosis	[107]
31	APAP-induced acute liver injury in male ICR mice	In vivo	200, 400 and 800 mg/kg for 10 days	↑ T-SOD, CAT, GSH ↓ Keap1, Nrf2, MDA, ALT, AST, AKP, TBIL, γ-GT	Puerarin protects against acetaminophen-induced oxidative damage in liver	[26]
	APAP‐induced HepG2 cell	In vitro	0, 3.75, 7.5, 15, 30, 60, 120 μmol/L for 24 h			
32	LPS/D-Gal-induced liver injury in male C57BL/6 mice	In vivo	200 mg/kg i.p	↑ LC3B-II/I, Beclin-1, SOD ↓ ALT, AST, p62, IL-1β, iNOS, MCP-1, RANTES, MDA	Protective role of puerarin on LPS/D-Gal induced acute liver injury via restoring autophagy	[24]
33	LPS/D-Gal-induced liver injury in male C57BL/6 mice	In vivo	25, 50 or 100 mg/kg	↑ ZEB2 ↓ ALT, AST, IL-1β, TNF-α, IL-6	Puerarin prevents acute liver injury	[23]
	LPS-induced L-02 cells	In vitro	10, 20, 30, 40 μM for 24 h			

### Effects of puerarin on acute liver injury

Acute liver injury (ALI) refers to sudden liver cell damage and liver dysfunction caused by various factors within a short period [19]. Viral infections, drug toxicity, and ischemia–reperfusion are common predisposing factors for ALI [20].

The inflammatory response plays a vital role in the pathogenesis of ALI [21]. As a transcription factor, zinc finger E-box binding homeobox 2 (ZEB2) includes multiple functional domains that interact with kinds of transcriptional co-effectors. Inflammatory cytokine production and epithelial-to-mesenchymal transition (EMT) can be mediated by ZEB2 [22]. Yang et al. [23] experimentally demonstrated that puerarin could prevent the activation of proinflammatory factors and attenuate LPS/D-Gal-induced liver injury by increasing the expression level of ZEB2, which in turn blocked the activation of the NF-κB signaling pathway in the liver. Meanwhile, puerarin has been shown to prevent LPS/D-Gal-induced ALI in mice, potentially through mechanisms related to autophagy activation and apoptosis inhibition [24].

Acetaminophen (APAP) is a commonly used medication for reducing fever and relieving pain, with significant antipyretic and analgesic effects. However, in clinical practice, excessive use of APAP has become a major cause of drug-induced liver injury (DILI) [25]. The active metabolites produced from APAP metabolism can lead to oxidative stress in liver cells. Zhou et al. [26] demonstrated that puerarin can alleviate oxidative stress and improve APAP-induced liver damage by inhibiting Keap1 and regulating the nuclear translocation of Nrf2.

### Effect of puerarin on metabolic dysfunction-associated steatotic liver disease

Non-alcoholic fatty liver disease (NAFLD) is being gradually replaced by metabolic dysfunction-associated steatotic liver disease (MASLD) because it does not accurately summarise the disease [27]. The “two-hit” hypothesis has been used in previous studies to explain the pathogenesis of MASLD. According to this, hepatic accumulation of lipids due to a sedentary lifestyle, a high fat diet, obesity and insulin resistance, acts as the first hit, sensitizing the liver to further insults acting as a “second hit”. The “second hit” activates inflammatory cascades and fibrogenesis [28]. With the gradual deepening of research, some scholars believe that the “multiple parallel hits” hypothesis can better explain the pathogenesis of MASLD, and these hits include insulin resistance, lipid metabolism disorders, oxidative stress, mitochondrial dysfunction, proinflammatory cytokines, immune responses, and gut flora disorders [29, 30]. However, MASLD remains a major public problem due to the lack of an effective treatment.

#### Puerarin improves MASLD by regulating lipid balance

Peroxisome proliferator-activated receptors (PPARs) are classified as members of the transcription factor nuclear receptor family and can be activated by a wide range of fatty acids and their derivatives [31]. PPARs consist of three main members, PPAR-α, PPAR-β/δ, and PPAR-γ [32]. PPAR-α is a nuclear receptor involved in the regulation of lipid metabolism and energy homeostasis, and it can be triggered by fatty acids and their by-products [33]. One study demonstrated that puerarin increased the expression level of PPAR-α in oleic acid (OA)-treated HepG2 cells [34]. Another study was the first to show the effect of puerarin on PPAR-γ, indicating that puerarin promotes fat metabolism and energy expenditure, thereby inhibiting fat accumulation [35].

Sirtuin 1 (SIRT1) is a crucial factor in regulating energy metabolism, particularly in the modulation of lipid and glucose metabolism in liver cells [36]. In HepG2 cells, puerarin significantly increased SIRT1 protein expression in a concentration- and time-dependent manner [37]. SIRT1 acts as an upstream regulator in the LKB1/AMPK signal transduction axis [38]. AMPK, a key energy sensor in cells, regulates the production of triglycerides (TG) and cholesterol (TC). The phosphorylation of AMPK can reduce the expression of lipogenic genes mediated by free fatty acids and decrease hepatic lipid accumulation [39]. Increased phosphorylation of AMPK promotes the phosphorylation of sterol regulatory element binding protein-1c (SREBP-1c) [40]. The transcription factor SREBP-1c is a master regulator of adipogenesis, involved in the transcriptional activation of genes encoding rate-limiting enzymes in adipogenesis, such as fatty acid synthase (FAS), acetyl-CoA carboxylase (ACC), and stearoyl-CoA desaturase 1 (SCD1) [41]. Research has demonstrated that puerarin can reduce lipid accumulation, leading to decreased mRNA expression of lipogenic genes such as SREBP-1c, FAS, and SCD-1, while increasing phosphorylation of AMPK and ACC in HepG2 cells [42].

Zheng et al. [43] demonstrated that puerarin has a therapeutic effect on MASLD by improving leptin signaling through the JAK 2/STAT 3 pathway. Leptin is a protein belonging to the adipokine family [44], which is mainly synthesized in adipocytes and plays a role in appetite suppression, promotion of energy expenditure, and regulation of glucose and lipid metabolism. However, obese individuals often develop leptin resistance, leading to leptin dysfunction [45].

#### Protective effects of puerarin on inflammation and mitochondrial homeostasis

The development and progression of inflammation is an important factor in many liver diseases. Hepatic inflammation is considered an important factor in accelerating the progression of simple fatty liver disease (SFLD) to NASH [46]. Specifically, the intake of puerarin reduces the serum and hepatic levels of inflammatory factors such as IL-18, IL-1β, and TNF-α [47].

Hepatocytes carry a large number of mitochondria, which are used to provide energy and regulate liver function [48]. Structural and functional alterations in mitochondria are critical for the development of MASLD. Structural alterations include depletion of mitochondrial DNA (mtDNA) and changes in morphology and ultrastructure, whereas functional alterations include defects in mitochondrial β-oxidation and respiration [49, 50]. These functional alterations lead to decreased ATP levels, ROS leakage, and excessive fat deposition [49]. Mitochondrial dysfunction is a major initiator of oxidative stress and plays a crucial role in the pathogenesis of MASLD [51]. Puerarin restores ATP, mtDNA, complex I and II activities in HFHS-fed mice and prevents HFHS-induced MASLD by promoting mitochondrial homeostasis [52]. Meanwhile, serum and liver levels of antioxidant markers SOD, GSH-Px, and CAT were significantly increased after puerarin administration [47].

#### Puerarin improves MASLD by reducing insulin resistance

Insulin resistance is an important feature of MASLD, and the relationship between the two is closely interconnected [29]. Insulin has the effect of inhibiting adipose tissue lipolysis. In the state of insulin resistance, enhanced adipose tissue lipolysis leads to a higher flux of free fatty acid into the liver, resulting in hepatic fat accumulation [28]. Studies have shown that puerarin ameliorates insulin resistance by increasing GLUT4 mRNA expression and activating the PI3K/Akt pathway [42].

### Protective effects of puerarin on alcohol-related liver disease

ALD is a chronic liver disease caused by excessive alcohol intake, which usually begins with alcoholic fatty liver disease. With continued drinking, liver cell metabolism becomes disrupted, leading to intracellular lipid accumulation. This can progress to more severe forms of liver damage, including alcoholic hepatitis, liver fibrosis, cirrhosis, and even hepatocellular carcinoma (HCC) [53–55]. According to the World Health Organisation (WHO) [56], about 2 million people die of liver disease each year, 50% of which are due to ALD, which remains a major public health problem due to the lack of efficient treatment. In recent years, many studies have found that puerarin has various degrees of therapeutic effects on ALD.

#### Puerarin improves ALD through oxidative stress

Microsomal ethanol-oxidizing system (MEOS), one of the pathways of ethanol metabolism [57], consists mainly of cytochrome P450 enzymes located in the smooth endoplasmic reticulum (SER), including CYP2E1 and CYP3A, amongst others [58]. Long-term alcohol consumption can lead to excessive activation of CYP2E1, which may result in lipid peroxidation and cell damage [59]. After treatment with puerarin, the levels of endogenous CYP2E1, CYP1A2 and CYP3A were reduced in liver tissues, which attenuated hepatocellular injury [60]. Experiments by Liu et al. [8] showed that puerarin treatment significantly down-regulated the mRNA levels of CYP2Y3 and CYP3A65, homologs of CYP2E1 and CYP3A, in a model of alcohol-induced zebrafish larvae. Puerarin may alleviate the development of alcoholic liver injury by increasing the levels of the antioxidant systems, such as glutathione (GSH), glutathione peroxidase (GPX), superoxide dismutase (SOD), and catalase (CAT) [61–63]. In the human body, the presence of small amounts of free radicals usually maintains the redox balance [64]. When free radicals and reactive oxygen species (ROS) are produced in excess, alterations occur in proteins, lipids, and DNA cells [65]. Organisms have developed a variety of antioxidant defences to scavenge the build-up of peroxides in the liver to reduce liver damage caused by oxidative stress [7, 66]. In vivo, experiments showed that puerarin could effectively alleviate chronic alcohol-induced liver injury in mice via the antioxidant HO-1 [62].

#### Puerarin improves ALD by suppressing inflammation/modulating immune response

In addition to causing bacterial translocation and endotoxemia by damaging the intestinal mucosa, alcohol consumption also triggers the release of pro-inflammatory factors like TNF-α and IL-1β from the liver when lipopolysaccharide (LPS) binds to toll-like receptors and activates Kupffer cells and other intrinsic immune cells [67]. In rats fed the Liber-DeCarli liquid diet, puerarin attenuated pathological changes in intestinal microvilli and up-regulated the expression of ZO-1 protein, while down-regulating the expression of CD68, lipopolysaccharide-binding proteins, CD14, toll-like receptor 2 and toll-like receptor 4 proteins [54]. These indicators reflect that puerarin can ameliorate alcoholic liver injury by inhibiting intestinal leakage of endotoxin, Kupffer cell activation, and endotoxin receptors. Meanwhile, treatment with puerarin can down-regulate the levels of inflammatory factors TNF-α, IL-1β, and IL-6 [62, 68]. Glycogen synthase kinase-3β (GSK-3β) and NF-kB have important roles in the inflammatory response and are also key components of the intracellular signaling cascade [69]. The protective effect of puerarin was associated with the inactivation of the GSK-3β/NF-kB pathway, which was accompanied by a concomitant reduction in GSK-3β-triggered apoptosis and NF-kB-mediated ethanol-induced inflammatory response in rat hepatocytes [70]. The inflammatory protective mechanisms of puerarin also include the inhibition of pro-inflammatory mediators COX-2, and 5-LOX [71], which are critical enzymes involved in leukotriene B4 (LTB4) production [72].

#### Puerarin improves ALD by reducing steatosis

Hepatic steatosis is also widely recognized as the earliest and the most common response of the liver to acute or chronic alcohol exposure [73]. Steatosis is characterized by the accumulation of TG, TC and phospholipids in hepatocytes [74]. In puerarin-treated animals, puerarin also reduced serum TG, TC, and free fatty acid (FFA) concentrations and restored cell viability [54]. Puerarin has also been shown to be effective in inhibiting ethanol-induced elevation of serum low-density lipoprotein cholesterol (LDL-C) [62]. AMPK is a serine protein kinase that plays an important role in the regulation of lipid and glucose metabolism [8]. In a larval model of alcohol-induced zebrafish, puerarin regulated alcohol-induced hepatic steatosis and reduced lipid accumulation, such as total TG and TC, through the AMPKa-ACC pathway and the FASN target [8]. Meanwhile, there have also been *ex-vivo* and *in-vivo* experiments demonstrating that puerarin can reduce the level of SREBP-1c and attenuate alcohol-induced liver injury [68].

#### Puerarin improves ALD by regulating cell death and prosurvival pathways

##### Autophagy

Autophagy is a dynamic process that maintains cellular homeostasis by removing damaged macromolecules and organelles to promote cell survival [74]. It is involved in degrading excessive lipid accumulation in hepatocytes and maintaining hepatic lipid metabolic homeostasis. As a subtype of autophagy, lipophagy refers to the process of degrading lipids or lipid droplets within cells through the autophagic pathway. In this process, lipid droplets (LDs) in the cytoplasm are engulfed by autophagosomes and transported to lysosomes, where lipids are broken down by lysosomal acidic lipases, thus protecting the liver from alcohol-induced fatty degeneration [75]. AMPK is a key energy sensor that regulates cellular metabolism to maintain energy balance. Conversely, autophagy is inhibited by the mammalian target of rapamycin (mTOR) [76]. In hepatocytes treated with high doses of ethanol, puerarin restores autophagy through the AMPK/mTOR signaling pathway, thereby alleviating ethanol-induced lipid accumulation in the liver [53]. Chronic ethanol consumption not only reduces the number of lysosomes but also inhibits the formation of autophagosomes, as evidenced by decreased levels of LAMP1 and LC3II [75]. Puerarin can promote the autophagic process by upregulating LC3II levels [53]. Research by Zhang et al. [77] indicates that an acute-on chronic ethanol-drinking according to the Gao-binge model induced alcoholic hepatitis (AH) pathology and resulted in hepatic hyper-autophagy. However, MSNs@Pue administration (puerarin: 30 mM, 42 mg/kg; intravenously [i.v.]) improved this condition.

##### Apoptosis

For a long time, oxidative stress has been closely related to cell death [78]. ROS are primarily produced in mitochondria, where they can damage mitochondrial DNA (mtDNA), impair the function of the respiratory chain, induce mitochondrial permeability transition, lead to mitochondrial swelling and rupture, and ultimately cause hepatocyte death [64]. Apoptosis is a form of programmed cell death that plays a crucial role in development and tissue homeostasis [79]. GSK-3β regulates glycogen synthesis to control glycogen metabolism and affects mitochondrial permeability and the release of cytochrome C to modulate apoptosis [80]. In a chronic alcoholic liver disease model, puerarin treatment can reduce the expression of GSK-3β at the protein level in rats [70].

### Protective effects of puerarin on hepatic fibrosis

#### Inhibition of activation of hepatic fibrosis

Liver fibrosis is a pathological process, not a separate disease. Liver fibrosis is a reversible wound-healing response to acute or chronic hepatocellular injury, reflecting the balance between liver repair and scar formation [81]. Liver fibrosis is characterized by the overproduction and deposition of extracellular matrix (ECM) in the liver [82]. During liver fibrosis, collagen is a major component of the ECM and plays roles such as supporting cell migration and guiding tissue development [83]. In rats with CCl4-induced hepatic fibrosis, puerarin significantly reduced histopathological changes as well as collagen type I and type III collagen levels in liver tissues [84, 85]. It has been shown that puerarin reduces serum and liver tissue levels of Col III, laminin, hyaluronic acid, hydroxyproline (Hyp), type III precollagen (PCIII), and Col I, and reduces the ECM deposition in liver tissues [86].

Hepatic stellate cells (HSCs) are the primary mesenchymal cells in the liver, accounting for 15% of the total number of resident hepatocytes and play a key role in liver fibrosis [82, 87]. The activation of HSCs consists of two main stages: ①The initiation, or pre-inflammatory stage, refers to early changes in gene expression shortly after injury. ② The perpetuation relates to the maintenance of the activation phenotype corresponding to the development of fibrosis [88]. The initiation stage is triggered by-products of injured hepatocytes, signals from Kupffer cells and endothelial cells, as well as ROS and lipid peroxide exposure [89]. These stimuli contribute to the persistence of hepatic fibrosis. A study showed that puerarin could improve oxidative stress and liver function in CCl4-induced hepatic fibrosis rats by increasing the antioxidant SOD and decreasing MDA levels [85]. It has also been shown that the beneficial effects of puerarin on hepatic fibrosis are related to the inflammatory pathway driven by NF-κB [85, 90]. Meanwhile, puerarin exerts its protective effects in CCl4-induced hepatic fibrosis, possibly through the inhibition of PARP-1 and the subsequent attenuation of NF-κB, ROS production and mitochondrial dysfunction [90].

Differentiation and accumulation of HSCs are usually induced by profibrogenic mediators, such as TGF-β [91]. During fibrosis, HSCs activate and transform into myofibroblast-like cells (MFB), which proliferate and synthesize excessive levels of ECM [92]. Activated HSCs not only promote the synthesis and deposition of ECM components but also the expression of α-SMA [91]. Elevated levels of α-SMA expression are a marker of activation in the HSC model [93]. Puerarin intake can moderate the activation of HSCs by reducing α-SMA expression [90, 94]. In TAA-induced liver fibrosis in male SD rats, puerarin reduces the activation of HSCs and attenuates the level of ECM expression by inhibiting the TGF-β/ERK1/2 pathway during hepatic fibrosis [94]. PPAR-γ plays an important role in the stimulation of HSC-mediated fibrosis. GW570 (PPAR-γ agonist) effectively inhibits collagenI, smooth muscle α actin mRNA and protein expression, consistent with the inhibition of HSC activation [95]. A study showed that puerarin could inhibit ECM-driven proliferation and activation of hepatocytes, such as HSC, fibroblasts and Kupffer cells, by activating endogenous PPAR-γ expression [86].

#### Promotes regression of liver fibrosis

Regression of liver fibrosis is associated with the inactivation or apoptosis of HSCs and MFBs [96]. The extent of matrix-degrading activity is determined by the balance of matrix metalloproteases (MMPs) and tissue inhibitors of MMPs (TIMPs) [97]. When hepatic fibrosis occurs, activated HSCs secrete fibrillar (or scarring) collagen, which leads to fibrotic matrix deposition, and can express TIMPs, which inhibit matrix-degrading metalloproteinase activity MMPs. This alters the balance of substrate secretion and degradation in favor of mechanisms that accumulate [98, 99]. Treatment with puerarin reduced the activity of TIMP-1 and enhanced the expression of MMP-1 and MMP-2 in rats [86, 100]. Increased cell death in hepatocytes leads to fibrosis, and cell death in HSCs is an important mechanism for resolving hepatic fibrosis [96]. At the same time, Zhang et al. [101] demonstrated that puerarin, by repairing hepatic injury and inducing apoptosis of HSCs through BCL-2, can effectively reverse chemical hepatic fibrosis.

### Protective effect of puerarin on hepatocellular carcinoma

HCC is one of the most common malignant tumors globally, directly contributing to nearly one million deaths annually [102]. In 2020, approximately 900,000 people were diagnosed with liver cancer worldwide, and this figure is projected to rise to 1.3 million by 2040 [103]. Due to the limited treatment options for advanced liver cancer, there is an urgent need to develop novel drugs for treating HCC patients. Research by Zeng et al. [104] found that puerarin significantly inhibited the proliferation of HCC SMMC7721 cells in a dose-dependent manner and induced significant apoptosis.

Mitogen-activated protein kinases (MAPK) are a class of serine/threonine kinases that respond to a variety of extracellular stimuli and mediate signaling from the cell surface to the nucleus. There are three well-characterized MAPK, with differing terminal serine/threonine kinases- ERK1/2 and 5, C-Jun amino-terminal kinase (JNK 1, 2, 3) and P38 kinases [105]. All these MAPK signaling pathways are involved in the regulation of apoptosis, and abnormalities in these pathways can evade apoptosis. Puerarin treatment increased the phosphorylation levels of ERK1, JNK and p38 in SMMC-7721 cells, thereby inducing apoptosis of MAPK signaling pathway-regulated hepatocellular carcinoma cells in a dose-dependent manner [106, 107]. Catabolism of mitochondrial membrane potential (MMP) occurs during the early stages of the apoptotic process. During MMP catabolism, the mitochondrial membrane pore is opened, leading to a loss of MMP [108, 109]. The loss of MMP leads to an increase in the permeability of the mitochondrial membrane and the release of pro-apoptotic molecules, such as cytochrome c and mitochondria-derived caspases activating factors, which trigger apoptosis [110]. One study demonstrated after treating SMMC-7721 cells with puerarin for 24 h, there was a significant, dose-dependent depolarization of MMP. [106].

## Toxicology and clinical applications of puerarin

Generally, the toxic effects of puerarin were not apparent in rodents in vivo or in vitro at doses up to 250 mg/kg per day [111]. The most common adverse reaction to puerarin is fever, followed by drug-induced dermatitis and hemolytic reactions. Fever caused by puerarin injection is mainly because it can easily pass the blood–brain barrier and stimulate the hypothalamic thermoregulatory center. It may also be related to the toxicity and accumulation caused by long-term high-dose use [112]. It should be emphasized that puerarin is a phytoestrogen, and long-term use of estrogen can cause various complications, such as breast cancer [113]. Meanwhile, puerarin has some reproductive toxicity effects. Chen et al. [114] investigated the reproductive toxicity effects of 2.5, 5.0, and 10.0 mol/L puerarin, and found that puerarin could induce apoptosis in the inner cells in mouse blastocysts, resulting in decreased embryo development and survival. Overall, literature on the toxicity of pueraria is extremely rare, which on the other hand suggests that pueraria may be a relatively safe natural product. However, from the current study, toxicity evaluation on animals is still lacking. Therefore, there is an urgent need for rigorous toxicity experiments to more accurately evaluate the safety of puerarin to promote its safe and rational development and clinical application.

**Fig. 3:**
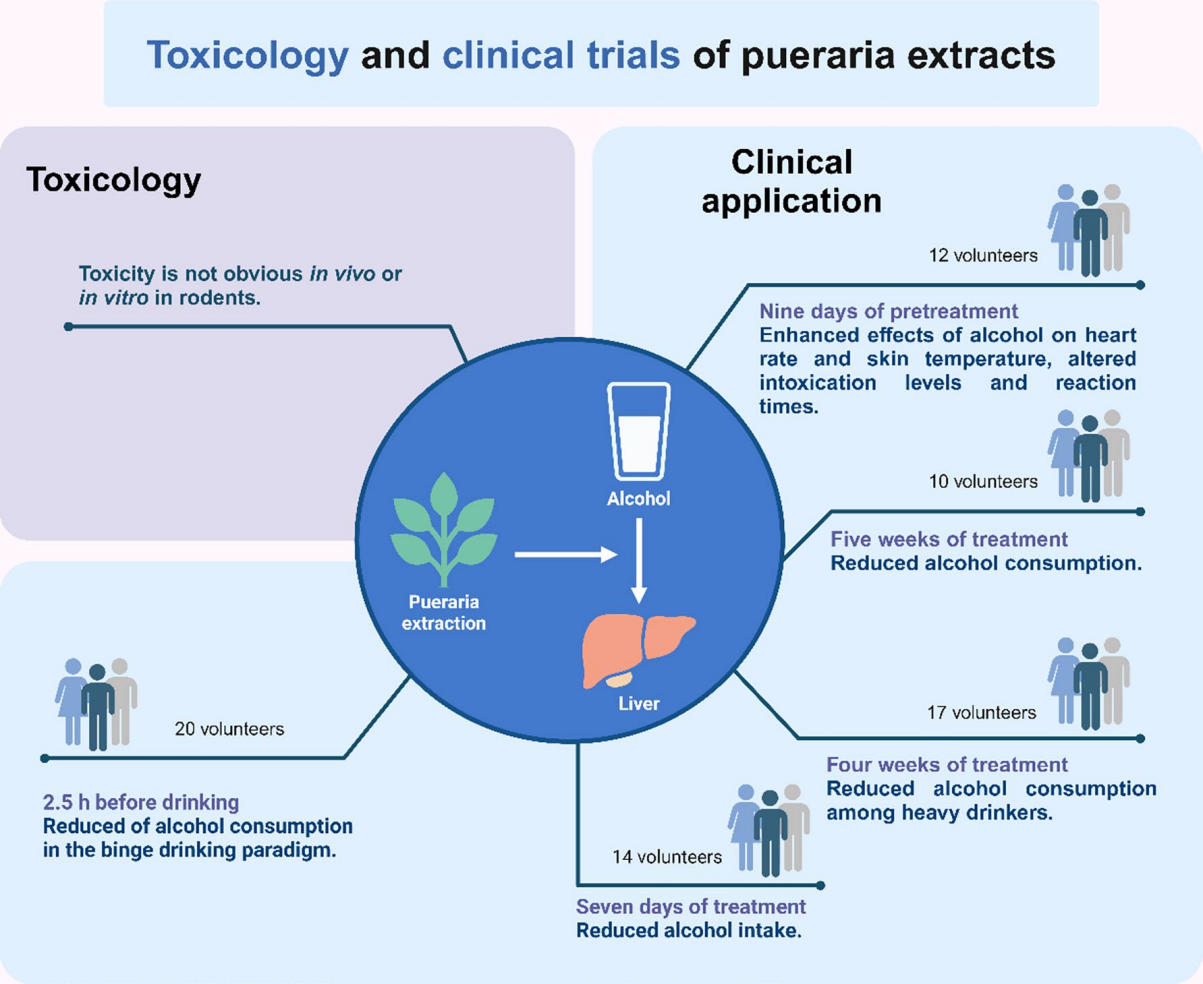
Toxicology and clinical trials of pueraria extracts

Currently, clinical research on puerarin for the treatment of liver disease is limited and singular. Most studies have used puerarin extract, administered in 500 mg capsules containing a sugar beet-based filler and three primary isoflavones: puerarin (19%), daidzin (4%), and daidzein (2%). Participants were given 2 capsules three times a day for a total daily dose of 750 mg of isoflavones [10, 115–118]. In a double-blind trial, experimenters treated with pueraria extract for nine days did not increase the intoxicating effects of acute alcohol [117]. Meanwhile, a four-week treatment study showed that pueraria extract significantly reduced weekly alcohol consumption with a reduction range of 34–57% [116]. In addition, treatment with pueraria extract not only affected the amount of alcohol consumed but also reduced the rate of drinking [10]. Based on these clinical trial results, pueraria extract effectively lowers both alcohol consumption and drinking rate with minimal side effects, as shown in Fig. 3.

## Pharmacokinetic study of puerarin

Pharmacokinetics is used to evaluate the safety and efficacy of drugs and guides the clinical use of puerarin. Studies have shown that puerarin has poor solubility due to its large conjugated skeleton, with a solubility of only 1.1 × 10–2%mol/L in water, resulting in low oral utilization of 7% [16, 119]. In an animal model, puerarin reaches maximum plasma concentration (Cmax) at 0.45–5.00 h post-dose, with an absorption half-life of 0.80–1.00 h and a distribution coefficient of 1.95 [120]. Puerarin is administered intravenously and is widely distributed in the hippocampus, mammary gland, liver, kidneys, spleen, stomach, tibia, and femur [121]. According to the biopharmaceutics classification system (BCS), puerarin can be classified as class IV due to its low solubility and limited intestinal permeability [122]. To improve the solubility of puerarin, co-solvents such as propylene glycol, ethylene glycol, and polyvinylpyrrolidone have been added to clinical injectable formulations. However, adverse drug reactions caused by co-solvents after intravenous injection, such as vascular irritation, fever, allergy and erythrocytolysis, are increasing year by year [12]. Therefore, the development of a new delivery system for puerarin is of great importance.

## A novel drug delivery system for pueraria

**Fig. 4:**
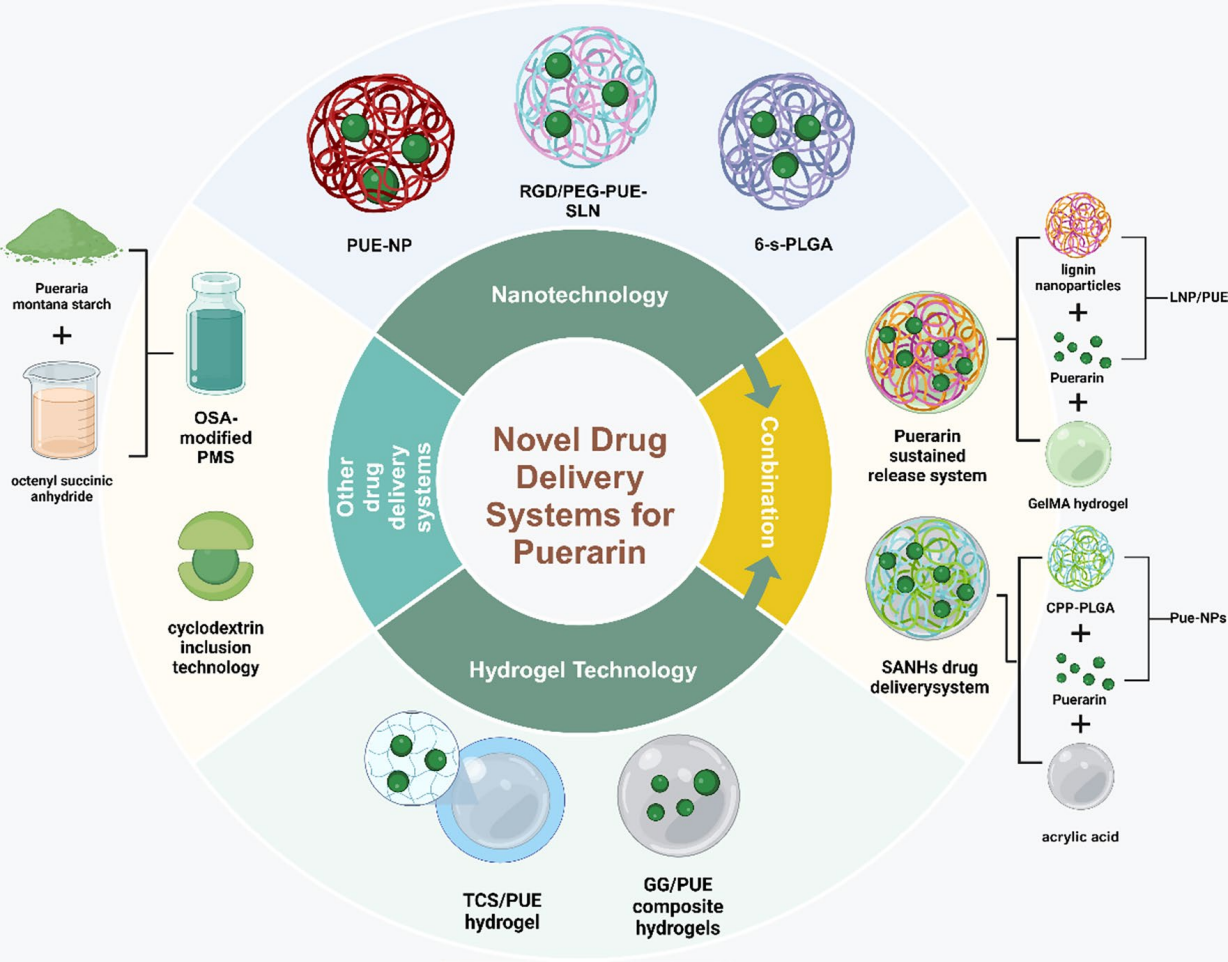
The novel drug delivery system of puerarin

The novel drug delivery system for puerarin is shown in Fig. 4.

### Nanotechnology

Currently, researchers are mainly using preparative techniques to enhance the oral bioavailability of puerarin, such as nanotechnologies and protein hydrogels.

Solid lipid nanoparticles and polymer nanoparticles are the focus of clinical drug delivery systems research [123]. Polylactic glycolic acid (PLGA) and PLGA-based composite nanoparticles have been widely used in targeting, imaging, and therapy, where PLGA can be completely degraded in an aqueous solution [124]. Researchers prepared Puerarin-PLGA nanoparticles (PUE-NP) by using PLGA nanoparticles as a synthetic material. PUE-NP delayed the release and metabolism of puerarin and increased its bioavailability in mice [125]. Dong et al. [126] prepared RGD (arginyl-glycyl-aspartic acid) modified and PEGylated solid lipid nanoparticles loaded with puerarin by the solvent evaporation method. After intravenous injection, the area under curve (AUC) of RGD/PEG-PUE-SLN was 176.5 (mg/mL h), compared to only 52.93 (mg/mL h) for free puerarin, significantly enhancing the bioavailability of puerarin. In another study, six-arm stellate poly (lactide)-ethyl lactate (6-s-PLGA) nanoparticles prolonged the in vivo half-life of geranylgeranyl and increased its bioavailability in the brain [127]. In addition, the preparation of ultra-small nanocrystals (less than 50 nm) by forming hydrogen bonds between puerarin and stabilizers can improve bioavailability and therapeutic efficiency [128].

### Hydrogel technology

Natural hydrogels are soft materials with high water content and a three-dimensional network structure, usually formed from hydrophilic natural polymers [129]. The construction of hybrid composite hydrogels can take advantage of the strengths of each component, so that the hybrid system exhibits an excellent combination of properties and improves its applicability [130]. Xu et al. [131] were the first to incorporate puerarin into gellan gum (GG) and explored the properties of GG/PUE composite hydrogels, including visual appearance, rheological properties, water distribution properties and structural properties, which laid the foundation for further applications. Yuan et al. [132] attempted to synthesize thiolated chitosan (TCS) and hybridize it with puerarin to prepare thiolated chitosan/puerarin composite hydrogel (TCS/PUE hydrogel) with pH/glutathione bi-responsiveness for drug delivery.

### Combined use of nanotechnology and hydrogel technology

However, geraniol hydrogels are very limited due to their weak thermal stability and lack of mechanical strength. Therefore, it is possible to combine nanotechnology with hydrogel technology. Hydrogels prepared based on poly acrylic acid (PAA) are uniquely hydrophilic, antimicrobial and biocompatible [133]. The researchers prepared puerarin-loaded nanoparticles (Pue-NPs) using the cell-penetrating peptide-poly (lactic-co-glycolic acid) (CPP-PLGA) as a drug carrier. They then employed the strategy of adding Pue-NPs into PAA to form hydrogels (PAA-Gel), developing a self-assembled nanocomposite hydrogels (SANHs) drug delivery system. Combining nanotechnology with hydrogels greatly improves the bioavailability of puerarin [134]. Pan et al. [135] used lignin nanoparticles (LNPs) as a scaffold for adsorbing puerarin and prepared puerarin-loaded LNPs (LNP/PUE). They then mixed LNP/PUE with GelMA hydrogel to develop a sustained-release system for puerarin. This system effectively improved blood perfusion in mice with hind limb ischemia.

### Others

Researchers have utilized modified-starch-stabilized Pickering emulsions containing microencapsulated puerarin, thereby enhancing its oral supplementation and accessibility [16]. Puerarin can also co-crystallize with l-proline, lurasidone hydrochloride (LH), and other adjuvants, improving the pharmacokinetics of oral drug preparations [119]. In addition, to improve the bioavailability of puerarin, cyclodextrin inclusion technology, solid dispersion technology, phospholipid complex technology, and other preparation technologies are also widely used [136].

## Discussion and outlook

In recent years, the incidence of liver diseases such as MASLD, ALD, and HCC has been gradually increasing with the improvement of living standards and the change of dietary structure and environment, which has brought a huge economic burden to the society. Currently, drugs for the treatment of liver diseases include Resmetirom [137], Entecavir [138], and Liraglutide [139] etc. These drugs are effective in specific conditions, but also face the challenges of side effects and high costs. In contrast, Chinese medicine offers a potential treatment for liver disease with its advantages of multiple pathways, multiple targets, fewer side effects and lower prices.

Puerarin is a natural component extracted from pueraria. Extensive research has demonstrated that puerarin possesses a wide range of pharmacological activities, such as reducing lipid accumulation, dilating blood vessels, inhibiting inflammation, and alleviating hangovers [11]. These properties suggest its potential applications in cardiovascular diseases, diabetes, kidney diseases, and liver diseases [12]. However, comprehensive reviews on the mechanisms of puerarin in liver diseases are still relatively limited. Therefore, this paper aims to summarize the mechanisms of puerarin in various types of liver diseases, providing a theoretical basis for further research and clinical applications. Based on our summary of the published literature, we believe that the recent research on puerarin has the following highlights. ① The pharmacological effects of puerarin in different liver disease models involve various aspects, including oxidative stress, inflammatory response, and lipid metabolism. This suggests that puerarin has potent biological activity and therapeutic potential to improve liver function and slow down disease progression. ② Pharmacokinetic studies reveal that puerarin is absorbed through multiple pathways and is widely distributed in tissues such as the hippocampus, mammary glands, liver, kidneys, spleen, stomach, tibia, and femur. This suggests that puerarin has favorable biodistribution characteristics. ③ The combination of puerarin with novel drug delivery systems has enhanced its bioavailability and therapeutic efficacy, overcoming the limitations of traditional administration methods. Overall, puerarin shows promising prospects for the prevention and treatment of liver diseases.

Although this review has summarized the therapeutic effects of puerarin on liver diseases, several limitations in current research still exist. ① The toxicological research on puerarin is still inadequate. As a major active component of traditional Chinese medicine, the interactions of puerarin with other drugs need further investigation. ② Most studies on puerarin’s treatment of liver diseases have mainly focused on in vivo and in vitro levels, and the clinical data are more limited. ③ The clinical application of puerarin with novel drug delivery systems remains underdeveloped. To overcome these limitations, future studies should focus on the following aspects. ① Additionally, research should explore its synergistic effects with other liver disease treatments and its compatibility with other natural substances or drugs. ② Establish high-quality clinical research protocols and conduct large-scale, multi-center, controlled trials to thoroughly evaluate the efficacy and safety of puerarin. ③ Pueraria, a traditional Chinese medicinal plant, enjoys high market acceptance. Currently, puerarin products available on the market include puerarin injection solutions and eye drops, which are used for cardiovascular diseases and dry eye syndrome, respectively. However, there are no puerarin-based drugs specifically for liver diseases. Developing such products could have significant clinical implications and a promising market potential.

In summary, while current research on puerarin is still in its early stages and its extensive pharmacological effects have yet to be fully integrated with clinical practice, puerarin's low cost, high safety, and notable efficacy provide a solid foundation for further research and development. The future development of puerarin holds both opportunities and challenges.

## Data Availability

No data was used for the research described in the article.

## Abbreviations

ACC: Acetyl-CoA carboxylase
ALD: Alcohol-related liver disease
ALI: Acute liver injury
APAP: Acetaminophen
AUC: Area under curve
BCS: The biopharmaceutics classification system
CAT: Catalase
CPP-PLGA: Cell-penetrating peptide-poly
DILI: Drug-induced liver injury
ECM: Extracellular matrix
EMT: Epithelial-to-mesenchymal transition
FAS: Fatty acid synthase
FFA: Free fatty acid
GPX: Glutathione peroxidase
GSH: Glutathione
GSK-3β: Glycogen synthase kinase-3β
HCC: Hepatocellular carcinoma
HFD: High fat diet
HSCs: Hepatic stellate cells
Hyp: Hydroxyproline
LDL-C: Low-density lipoprotein cholesterol
LH: Lurasidone hydrochloride
LNPs: Lignin nanoparticles
LPS: Lipopolysaccharide
LTB4: Leukotriene B4
MASLD: Metabolic dysfunction-associated steatotic liver disease
MAPK: Mitogen activated protein kinases
MEOS: Microsomal ethanol-oxidizing system
MFB: Myofibroblast
MMP: Mitochondrial membrane potential
MMPs: Matrix metalloproteases
mtDNA: Mitochondrial DNA
NAFLD: Non-alcoholic fatty liver disease
OA: Oleic acid
OSA: Octenyl succinic anhydride
PAA: Poly acrylic acid
PLGA: Poly lactic glycolic acid
PMS: Pueraria montana starch
PPARs: Peroxisome proliferator-activated receptors
PUE-NP: Puerarin-PLGA nanoparticles
ROS: Reactive oxygen species
SANHs: Self-assembled nanocomposite hydrogels
SCD1: Stearoyl-CoA desaturase 1
SER: Smooth endoplasmic reticulum
SFLD: Simple fatty liver disease
SIRT1: Sirtuin 1
SREBP-1c: Sterol regulatory element binding protein-1c
SOD: Superoxide dismutase
TC: Tholesterol
TCM: Traditional Chinese Medicine
TCS: Thiolated chitosan
TG: Triglycerides
TIMPs: Tissue inhibitors of MMPs
WHO: World Health Organisation
ZEB2: Zinc finger E-box binding homeobox 2
